# Miniature Microring Resonator Sensor Based on a Hybrid Plasmonic Waveguide

**DOI:** 10.3390/s110706856

**Published:** 2011-07-01

**Authors:** Linjie Zhou, Xiaomeng Sun, Xinwan Li, Jianping Chen

**Affiliations:** State Key Laboratory of Advanced Optical Communication Systems and Networks, Department of Electronic Engineering, Shanghai Jiao Tong University, Shanghai 200240, China; E-Mails: sunxiaomeng@sjtu.edu.cn (X.S.); lixinwan@sjtu.edu.cn (X.L.); jpchen62@sjtu.edu.cn (J.C.)

**Keywords:** surface plasmon resonance, resonators, sensors

## Abstract

We propose a compact 1-μm-radius microring resonator sensor based on a hybrid plasmonic waveguide on a silicon-on-insulator substrate. The hybrid waveguide is composed of a metal-gap-silicon structure, where the optical energy is greatly enhanced in the narrow gap. We use the finite element method to numerically analyze the device optical characteristics as a biochemical sensor. As the optical field in the hybrid micoring resonator has a large overlap with the upper-cladding sensing medium, the sensitivity is very high compared to other dielectric microring resonator sensors. The compactness of the hybrid microring resonator is resulted from the balance between bending radiation loss and metal absorption loss. The proposed hybrid microring resonator sensors have the main advantages of small footprint and high sensitivity and can be potentially integrated in an array form on a chip for highly-efficient lab-on-chip biochemical sensing applications.

## 1. Introduction

On-chip label-free biochemical sensors have attracted much research interest in recent years due to their small footprint and high sensitivity [1,2]. The miniaturization of sensors is of great importance for lab-on-chip sensing applications, as only a small amount of analyte is needed to accomplish the analysis. The miniature sensors can also be arranged in the form of an array to simultaneously sense different analytes. There are various approaches to realize on-chip optical sensors, including waveguide-based sensors [3–5], microring or microdisk-based sensors [6–11], photonic crystal resonance-based sensors [12–14], Mach-Zehnder or dual-slit interferometer-based sensors [15–17] and nanostructured plasmonic sensors [2,18–22].

The dielectric microresonators are very attractive as they have very high quality-factors (Q-factors) with very sharp resonance line-shapes, which can used to detect a small ambient refractive index change. However, to generate such high-Q resonances, the cavities cannot be very small as the associated radiation loss increases exponentially with a decreasing bending radius. Besides, for such microresonators, the optical energy is mainly localized in the dielectric core and only a very small amount of energy (in the form of evanescent field) is distributed outside in the cladding sensing medium, which ultimately limits their sensitivity. Although reducing waveguide dimensions or using slot waveguide structures in the microring resonators can improve the sensitivity [10,11], the low optical confinement of the waveguide requires a even larger bending radius to maintain a low loss.

On the other hand, waveguides based on surface plasmon resonances (SPPs) have a very small mode volume and a tight bending curvature, as the surface plasmon wave is generated via the coherent oscillation of free electrons at the metal-dielectric interface and thus it is tightly bound to the metal surface. However, they usually have a relatively high propagation loss compared to dielectric waveguides, because of the absorption loss in the metal material. Hybrid plasmonic waveguides, which combines the merits of the dielectric and plasmonic waveguides, have recently been widely investigated [23–25]. They have a high optical confinement beyond the diffraction limit while the propagation loss is relatively low.

Here, we propose to employ a hybrid plasmonic waveguide to form a compact 1-μm radius microring resonator for high-sensitivity refractive index sensing. The excitation of surface electron oscillation in the hybrid waveguide essentially renders the photons to bind more closely to the metal surface, and as a result, the radiation loss is reduced. Hence, the microring resonator formed by a hybrid plasmonic waveguide can in principle be very compact. In addition, the use of a slot waveguide structure makes the optical energy well-confined in the narrow slot, which enhances its overlap with the cladding medium and helps to improve its sensitivity. The high compactness and high sensitivity of the hybrid plasmonic microring resonator sensors make them suitable for point-of-care diagnostics in lab-on-chip sensing applications.

## 2. Device Structure and Working Principle

**Figure 1.**
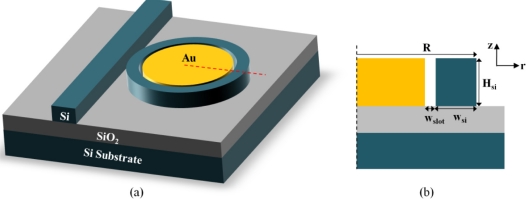
**(a)** Schematic structure of the proposed hybrid plasmonic waveguide-based microring sensor. **(b)** Cross-sectional view of the hybrid microring resonator with the geometric dimensions labeled in the figure.

Figure 1(a) shows the schematic of the proposed hybrid plasmonic waveguide-based microring resonator sensor. The microring resonator is composed of a silicon circular strip encompassing a metal disk in the center. Figure 1(b) shows the cross section of the microring resonator. The silicon ring radius is *R* (from the outer edge). The silicon strip has a width of *W_si_* and a height of *H_si_*. The metal layer has the same height with the silicon layer, and its separation from the silicon strip is *W_slot_*. The microring resonator is side-coupled with a regular silicon ridge waveguide for resonance excitation. The resonance transmission spectrum from the waveguide is tracked to monitor the cladding refractive index change.

The dielectric silicon photonic mode and the surface plasmonic mode are strongly coupled to form the hybrid mode. The coupling strength is affected by both the slot and the silicon strip widths. Due to the excitation of surface plasmon wave and the discontinuity of electric-field across the silicon strip sidewall, the electric-field is greatly enhanced in the narrow slot. As there is a large overlap between the waveguide mode and the cladding medium, a small refractive index change can result in a larger resonance wavelength shift, compared to regular microring sensors.

## 3. Performance Analysis

We use the finite element method (FEM) to numerically calculate the hybrid plasmonic waveguide mode. Gold is chosen as the metal layer, as it is biocompatible and not easily oxidized compared to silver. The refractive indices of the constitutive materials at 1,550 nm wavelength are *n_ox_* = 1.444, *n_si_* = 3.477, and *n_Au_* = 0.55 + 11.5*i* for oxide, silicon, and gold, respectively. The device upper-cladding sensing medium is assumed to be water solution with its complex refractive index *n_c_* = 1.33 +1.2e − 4*i* [26]. The water absorption loss is relatively small (∼0.0042 dB/μm) compared to the metal absorption loss and the hybrid waveguide propagation loss [Figure 3(b)], and hence its influence on the device performance can be neglected. We fix the waveguide height at *H_si_* = 250 nm, a typical dimension for silicon ridge waveguides. The bending radius is set at *R* = 1 μm.

Distinct from the straight waveguide, the bent waveguide in the hybrid microring resonator has a different electromagnetic field distribution with its mode profile pushed more outwards. To calculate the bent waveguide mode, we employ the conformal mapping to convert the bent waveguide to a straight waveguide [27]. The refractive index is no longer uniform in the converted waveguide, but instead, it increases exponentially towards the radial direction. In our FEM simulations, the subdomains in the waveguide cross section were partitioned into triangular mesh elements with quartic Lagrange functions. The grid size is set to be 10 times smaller than the dimensions of the corresponding regions and the maximum mesh element size is set to be 20 nm, such that accurate simulation results can be achieved within the allowed time and computational resources. Perfect matched layers (PMLs) were also added at the outer edges of the FEM simulation window to approximate an open geometry [28]. The PML was positioned 0.6 μm away from the waveguide edge. The PML width is 1 μm and the grid size inside the PML is 50 nm. The PMLs have uniform, anisotropic, and complex dielectric constants so that the reflections at the edges were eliminated and radiated light was attenuated inside the PMLs. The dielectric constants of the PMLs were chosen to match those at the edge of the equivalent straight waveguide, and the absorption constant and width of the PML were chosen to ensure light only propagates several mesh elements before being completely absorbed. Perfect electric conductors were used at all boundaries. The calculated bent waveguide mode is composed of a guided part and a leaky part, and hence, both the waveguide propagation constant and the loss can be obtained simultaneously from the FEM simulations.

**Figure 2.**
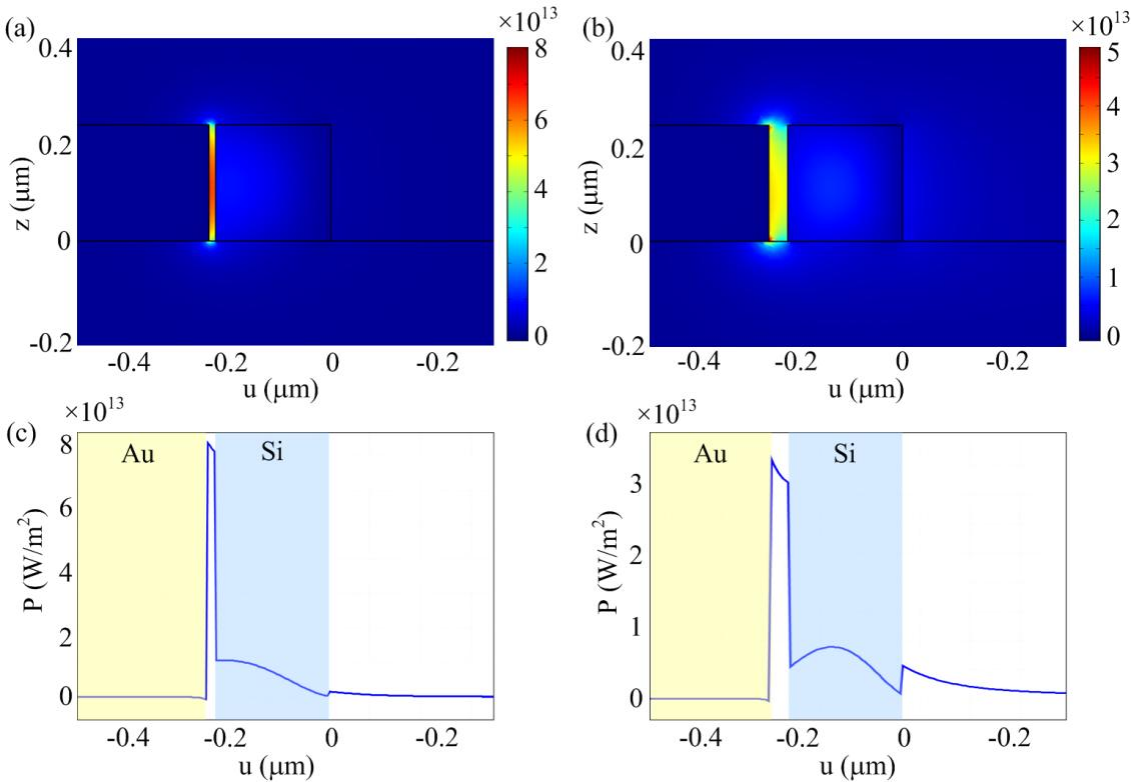
**(a)** and **(b)** Average optical power flow in the propagation direction for (a) *W_slot_* = 10 nm and (b) *W_slot_* = 30 nm. **(c)** and **(d)** are the corresponding optical power flow density along a lateral line in the middle of the waveguide. Total power flow is assumed to be 1 W. *u* is the transformed axis after conformal mapping.

Figure 2(a,b) shows the optical power flow density contour plots in the cross section of the bent hybrid plasmonic waveguide for two slot widths of 10 nm and 30 nm. The silicon strip width is 200 nm. The corresponding optical power flow density profiles along a lateral line in the middle of the waveguide are shown in Figure 2(c,d). It can be seen that the central metal block can attract the optical power to the narrow slot region. The closer the silicon strip approaches the metal block, the more the optical power is confined near the metal surface. Note that the evanescent field outside the microring outer rim is weaker for the 10 nm slot case than that for the 30 nm slot case. Therefore, the addition of the metal block reduces the mode power radiation leakage, making the low loss propagation possible even for a highly curved waveguide.

**Figure 3.**
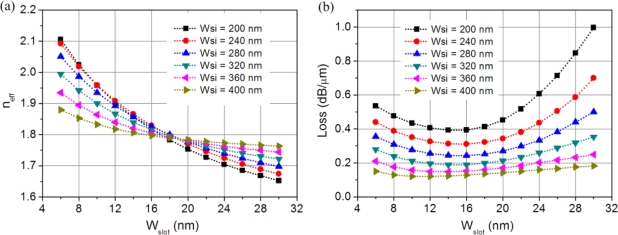
**(a)** Effective refractive index (real part) and **(b)** propagation loss of the hybrid microring waveguide versus slot width.

Figure 3(a,b) shows the bent waveguide effective refractive index and the propagation loss change as function of slot width for various silicon strip widths. The effective refractive index and the propagation loss values are referred to the microring resonator outer rim. The slot width has a large effect on the hybrid mode effective index and the propagation loss, especially for narrow silicon strips. When the slot width decreases, the optical power outside the waveguide is attracted to the silicon core, causing the effective index to increase. For the propagation loss, however, when the slot width reduces, it first decreases and then increases if the silicon strip is not too wide. There are two sources for the bent waveguide loss: the radiation loss and the metal absorption loss, with the former increasing and the latter decreasing with the slot width. Their competition ultimately determines the bent waveguide loss. Therefore, there is an optimum slot width where the bent waveguide loss reaches the minimum. For example, when the silicon strip width is 200 nm, the optimum slot width is ∼16 nm. The excitation of surface plasmon waves can reduce the otherwise large bending loss suffered in a regular dielectric waveguide, which is more obvious for narrow silicon waveguides. The implication is that the low-loss propagation can be extended to a highly-curved waveguide, which makes very compact microring resonators possible.

**Figure 4.**
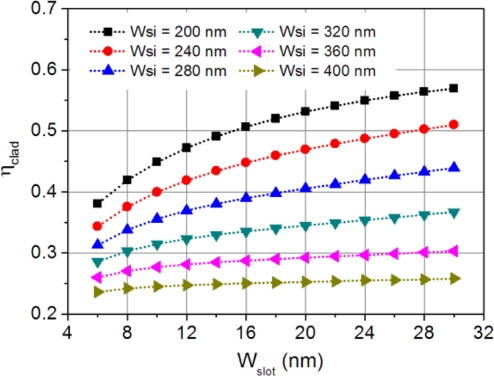
Upper-cladding layer confinement factor changes as a function of slot size for various silicon widths.

As the overlap between the optical field and the analyte solution determines the sensitivity of the sensor, we define an upper-cladding confinement factor *η_clad_* to describe the overlap level. The upper-cladding confinement factor can be expressed as:

(1) $$\eta_{\mathrm{clad}}=\frac{\text{Re}\left(\iint_{S_{\mathrm{clad}}}\vec{E}\times \vec{H}\cdot \hat{z}\mathrm{dS}\right)}{\text{Re}(\iint_{S}\vec{E}\times \vec{H}\cdot \hat{z}\mathrm{dS})},$$

where *E⃗* and *H⃗* are the electric and magnetic fields, *S_clad_* indicates the upper-cladding layer region (including the slot), and *S* is the hybrid plasmonic waveguide cross-section. The dependence of the upper-cladding confinement factor on the slot and the silicon strip widths is shown in Figure 4. A large slot gap and a narrow silicon width push the optical energy into the upper-cladding layer, which increases its overlap with the analyte solution. For example, when the silicon width is 200 nm and the gap is >16 nm, more than half of the optical energy is distributed in the upper-cladding layer.

The sensitivity *S* is defined as the ratio between the resonance wavelength shift and the cladding refractive index change, *i.e.*, *S* ≡ *dλ/dn_c_*, which is a key parameter to describe the sensor performance. The resonance wavelength *λ* satisfies the resonance condition *mλ* = *n_eff_L* (*m* is an integer), where *L* is the circumference of the microring resonator. Taking into account the wavelength dispersion of *n_eff_*, we can express the sensitivity as:

(2) $$S=\frac{\lambda }{n_{g}}\frac{\partial n_{\mathrm{eff}}}{\partial n_{c}},$$

where *n_g_* is the group refractive index given by:

(3) $$n_{g}=n_{\mathrm{eff}}-\lambda \frac{\partial n_{\mathrm{eff}}}{\partial \lambda }.$$

**Figure 5.**
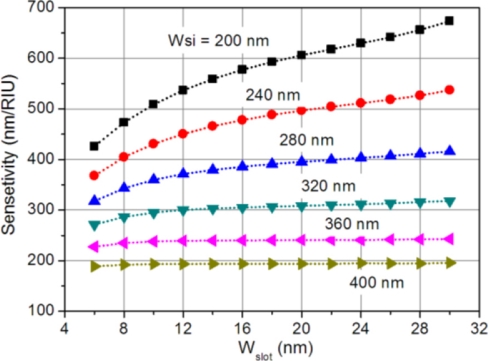
Sensitivity changes as a function of slot size for various silicon widths.

Hence, to get a high sensitivity, the change rate of the effective refractive index to the cladding refractive index should be large, or in other words, the cladding confinement factor should be large. Figure 5 shows the sensitivity changes as a function of slot width for various silicon widths. The change trend is similar to that of the cladding confinement factor in Figure 4. To obtain a high sensitivity, the slot should be large and the silicon strip should be thin.

**Figure 6.**
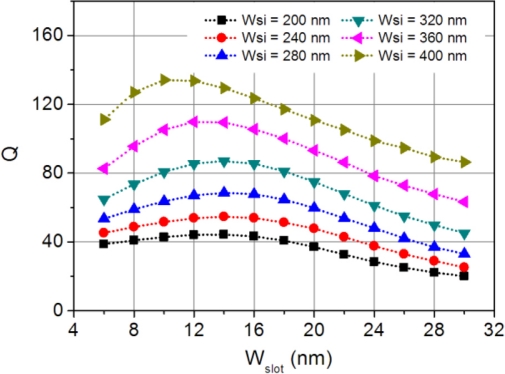
Resonance Q-factor of the hybrid microring resonator changes as a function of slot size for various silicon widths.

The sensitivity is used to measure the resonance wavelength shift in response to the cladding index change. It is independent of the resonance spectral profile. However, the detection limit *DL*, which is defined as the minimum refractive index change in the sensing medium that can be detected by the sensor system, is proportional to the resonance linewidth Δ*λ* or inversely proportional to the resonance Q-factor. The Q-factor is determined by the resonator loss (intrinsic Q-factor) as well as the coupling loss to the straight waveguide (external Q-factor). For the microring resonator working at the critical coupling regime, the Q-factor can be expressed as [29]:

(4) $$Q=\frac{\lambda }{\Delta \lambda }\approx \frac{\pi n_{\mathrm{eff}}L\sqrt{1+a^{4}}}{\sqrt{2}\lambda (1-a^{2})},$$

where *a* is the electric-field round-trip transmission coefficient in the resonator. Figure 6 plots the Q-factor variation with the slot width. It has an inverse change trend with respect to the loss curves in Figure 3(b). Although the Q-factor for this hybrid microring resonator is around 10^2^, it is much larger than that of microring resonators composed solely by dielectric or plasmonic waveguides with the same cavity size. When the slot is very wide, the hybrid microring resonator regresses to a dielectric microring resonator; and on the contrary, when the slot becomes very narrow, it resembles a plasmonic microring resonator. The combination of the dielectric and plasmonic guiding future of the hybrid waveguide increases the resonance Q-factor.

**Figure 7.**
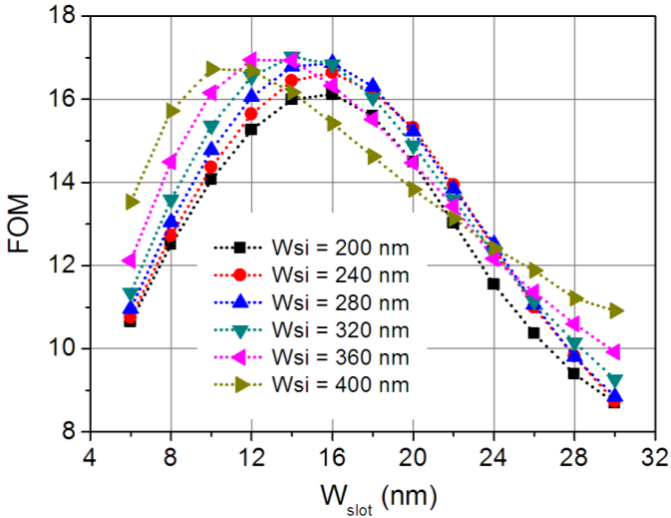
Figure of merit (FOM) of the microring sensor changes as a function of slot size for various silicon widths.

As can be seen from Figures 5 and 6, the sensitivity and Q-factor have a distinct change trend and they cannot be obtained simultaneously. A high sensitivity features a large resonance wavelength shift for a given index change, but it is more difficult to detect a small refractive index change. On the contrary, a high Q-factor improves the detection limit but reduces the resonance wavelength shift. To properly evaluate the sensing performance of the proposed hybrid microring resonator sensor, a figure of merit (FOM) can be defined as [8,22]:

(5) $$\mathrm{FOM}=\frac{S}{\Delta \lambda }=\frac{S}{\lambda }Q,$$

which equals the number of resonance linewidth shift in response to a unit cladding refractive index change. Figure 7 shows the FOM changes with the slot width at various silicon strip widths. The maximum achievable FOM is ∼17, which occurs at *W_slot_* = 10 to 16 nm depending on the silicon strip width. Note that, as the slot serves as a host for the biochemical analyte, only those with dimensions smaller than the slot can be infiltrated into the slot to induce a resonance wavelength shift.

## 4. Discussion

In the above analysis, we use conformal mapping and FEM to calculate the bent waveguide leaky eigenmodes with the wavelength fixed at 1,550 nm. In practice, the resonance of the hybrid microring resonator does not always occur at 1,550 nm, and instead, it is determined by the resonance azimuthal mode number *m*. For more precise analyses of the resonance mode of the hybrid microring resonator, the wavelength should be set to its resonance wavelength. Using the fixed wavelength, however, it is more convenient for us to analyze the device performance change trend with a particular geometric parameter (the wavelength dispersion of the hybrid waveguide mode is decoupled), which can provide a guideline for the device design. On the other hand, the resonance wavelength can be set to 1,550 nm by slightly adjusting the microring radius.

We assume the hybrid microring resonator works at the critical coupling regime, as it is corresponding to the highest resonance extinction ratio, which, as a result, leads to a maximized signal-to-noise ratio. The critical coupling can be achieved by optimizing the straight waveguide width and its separation with the microring resonator so that the coupling strength and the micoring resonator loss are balanced [29]. Given the high loss (low-Q) of the hybrid microring resonator, it may be still challenging to reach the critical coupling by manipulating the straight access waveguide. Another effective approach that can effectively increase the coupling strength is to wrap a curved access waveguide around the microring resonator to increase the coupling interaction length.

The detection limit is a critical parameter to assess the sensor performance. It is decided by the resonance Q-factor as well as the detector system noise level (coming from temperature fluctuation, light source, photodetector *etc.*). The detection limit can be expressed as:

(6) $$\mathrm{DL}=\frac{f\lambda }{\mathrm{QS}}=\frac{f}{\mathrm{FOM}},$$

where *f* is a factor describing the fraction of resonance linewidth Δλ that can be resolved by the detection system (*f*Δλ is thus the resonance wavelength resolution). In practice, the resonance wavelength resolution is quite dependent on the overall signal-to-noise ratio in the detection system. Following the resolution analysis in [11], a reasonable value for *f* is around 1/400. Thus, the detection limit for our device at its optimal performance (FOM = 17) is ∼1.5 × 10^−4^ RIU.

The hybrid microring sensor can be fabricated on a silicon-on-insulator (SOI) substrate by using conventional fabrication techniques. First, the access waveguide and the silicon microring strip are defined by e-beam lithography (EBL) and then etched by reactive ion etching (RIE). Then, the device is put into an oxidation furnace to form a thin layer of oxide with its thickness equal to desired slot width. Gold film is deposited by using e-beam evaporation and patterned to fill only inside the microring by using a liftoff process. To flatten the metal layer to the same height with the silicon waveguide, a chemical-mechanical polishing (CMP) process can be employed. After CMP, the microring inner sidewall oxide is uncovered and can be etched away by using a selective chemical wet etch. It can be seen that, by using an oxidation process, the metal block and the silicon strip can be auto-aligned with a uniform separation, which is the critical step to make the device operational. Besides, the oxidation process can help to reduce the sidewall roughness and improve the resonance quality factor. It should be noted that the two lithography patterns do not need to align very accurately as long as the metal covers the microring inner rim while leaving the outer rim uncovered.

The performance of our proposed sensor in terms of FOM (FOM ≈ 17) is inferior to conventional dielectric microring resonators, which usually have high-Q resonances and thus high FOM values (FOM ≈ 4,500) [9]. The FOM of our device is also smaller than those of slot and hybrid SPP ring resonator sensors, which have typical FOM values of 58 and 77 [8,11]. Although our device is not superior to other microring resonator-based sensors in terms of FOM, the main advantages of our device are its high sensitivity and compact size. A typical sensitivity of our device is 580 nm/RIU (*W_slot_* = 16 nm and *W_si_* = 200 nm), much larger than 163 nm/RIU for the dielectric microring sensor [9]. Using a slot waveguide structure in microring resonators can improve the sensitivity to 298 nm/RIU [10,11], and yet it is still smaller than that of our device. Moreover, the radius of the slot microring resonator cannot be very small, typically around 5 μm [11], and thus, in terms of device footprint, our device is more than one order smaller. The small footprint of our sensor can help to reduce the amount of analyte needed for sensing measurements. Reducing the device dimension is also an important step towards large-scale integration of this kind of miniature sensors onto a lab-on-chip sensing system.

On the other hand, in terms of device footprint, our device is larger than surface plasmon resonance (LSPR) sensors (typically 100 nm in size). However, the performance (FOM and sensitivity) of our proposed device is superior to that of LSPR sensors. For example, the FOM of a LSPR sensor based on a single silver triangular nanoprism is only 3.3 with a sensitivity of 205 nm/RIU [22]. In addition, LSPR sensors lack the potential for large-scale on-chip integration, as it is difficult to couple light to LSPR sensors and difficult to integrate LSPR sensors with other optoelectronic devices, which is a desirable feature for future lab-on-chip integrated system.

## 5. Conclusions

We have proposed a miniature biochemical sensor based on a 1-μm-radius hybrid plasmonic microring resonator. The hybrid mode in the microring resonator waveguide is formed by mutual coupling between the photonic mode in the silicon waveguide and the plasmonic mode at the metal surface. Due to the excitation of surface plasmon wave, the mode power is pulled inside towards the metal surface which reduces the radiation loss and results in a low-loss hybrid plasmonic mode. The hybrid microring sensor exhibits a relatively high sensitivity, thanks to the large overlap between the hybrid mode and the cladding sensing medium. The sensing performances of our proposed device were investigated by numerical simulations and their dependence on the geometric parameters was analyzed in details. A typical sensitivity of 580 nm/RIU was achieved with a FOM of 16. Given its small footprint and high sensitivity, the sensor can find potential applications in label-free lab-on-chip biochemical diagnoses.
